# Characteristics of Patients with Sarcoidosis with Emphasis on Acute vs. Chronic Forms—A Single Center Experience

**DOI:** 10.3390/jpm14060616

**Published:** 2024-06-08

**Authors:** Mihailo Stjepanovic, Nikola Maric, Slobodan Belic, Jelena Milin-Lazovic, Natasa Djurdjevic, Jelena Jankovic, Masa Petrovic, Jovan Peric, Ivan Tulic, Jelena Cvejic, Spasoje Popevic, Sanja Dimic Janjic, Violeta Mihailovic Vucinic

**Affiliations:** 1 Clinic for Pulmonology, University Clinical Center of Serbia, 11000 Belgrade, Serbia; nikolamaric1994@gmail.com (N.M.); belicslobodan@hotmail.com (S.B.); natalidjurdjevic@yahoo.com (N.D.); jjelena1984@gmail.com (J.J.); cvejicjelena03@gmail.com (J.C.); spasapop@gmail.com (S.P.); sanjadimicjanjic@gmail.com (S.D.J.); 2 Medical Faculty, University of Belgrade, 11000 Belgrade, Serbia; milinjelena@gmail.com (J.M.-L.); 5rovicmasa@gmail.com (M.P.); violetavucinic@gmail.com (V.M.V.); 3 Institute for Medical Statistics and Informatics, Faculty of Medicine, University of Belgrade, 11000 Belgrade, Serbia; 4 Institute for Cardiovascular Diseases “Dedinje”, 11000 Belgrade, Serbia; 5 Center for Anesthesiology and Resuscitation, University Clinical Center of Serbia, 11000 Belgrade, Serbia; jovan.peric994@gmail.com; 6 Clinic for Orthopedic Surgery and Traumatology, University Clinical Center of Serbia, 11000 Belgrade, Serbia; tulic95ivan@gmail.com

**Keywords:** sarcoidosis, epidemiology, chitotriosidase

## Abstract

Sarcoidosis is a granulomatous disease of unknown etiology that can affect almost any organ. Although the acute form can have spontaneous regression, a certain number of patients can have a chronic form, which leads to an increase in mortality and a decrease in the quality of life. Considering that the risk factors are still unknown, we wanted to compare the characteristics of patients with acute and chronic forms of sarcoidosis in Serbia in order to determine significant differences between them with hopes of contributing to everyday clinical practice. A total of 2380 patients treated in our clinic were enrolled in this study. They were separated into the following two groups: 1126 patients with acute form and 1254 patients with chronic form. They were further compared by gender, smoking status, radiological status, exposition, biomarkers for sarcoidosis, organ involvement, and other comorbidities; the distribution of patients according to regions of Serbia was also noted. Statistical significance was found in radiological findings (*p* < 0.001), biomarkers (calcium in 24 h urine *p* < 0.001; chitotriosidase *p* = 0.001), and the affliction of organs (*p* < 0.001). The differences noted in this paper could help improve our understanding of this disease.

## 1. Introduction

Sarcoidosis is a multisystem disease, first described in the late 19th century, characterized by the presence of non-necrotizing granulomas in different tissues, most commonly in the lungs and associated lymph nodes [1]. The term “sarcoidosis” stemmed from Caesar Boeck’s report in which he described benign skin lesions as “sarkoids” due to their close resemblance to sarcomas [2]. Sarcoidosis is relatively rare, with an estimated incidence of between 2.3 and 11 per 100,000 individuals per year and a proportionally higher incidence among African Americans and Scandinavians compared to the rest of the Caucasian population [3,4]. When observing sarcoidosis across Europe, a higher incidence was observed in the northern regions, with incidence rates of 64/100,000 individuals in Sweden and 43/100,000 individuals in Germany. In contrast, counties in Southern Europe tended to have a lower incidence; for example, Italy had an incidence of 9 per 100,000 individuals [4]. In Serbia (seven million inhabitants), the prevalence of sarcoidosis was previously estimated to be 16.5 per 100,000 individuals; however, it should be noted that Serbia does not possess a central register of sarcoidosis patients.

Even though the cause of this disease remains unknown, our understanding of the underlying mechanisms that lead to granuloma formation, such as genetic susceptibility and environmental factors, is increasing [5,6]. For example, nicotine has been suggested to play a potential protective role in sarcoidosis, evidenced by a lower incidence observed in a smoker population [2]. In contrast to nicotine, exposure to insecticides, mold, and air condition devices was noted as a potential risk, as their presence was noted in patients with sarcoidosis [7].

Sarcoidosis can manifest as either acute or chronic. The most common form is acute, which generally has a good prognosis, with patients often achieving complete remission within two years. A notable form of acute sarcoidosis is called Löfgren syndrome, characterized by erythema nodosum, arthritis, and bilateral lymphadenopathy [8]. Another form of acute sarcoidosis, Heerfordt–Waldestrom, involves parotid gland enlargement and facial nerve palsy [9]. Chronic sarcoidosis, on the other hand, is characterized by the persistence of symptoms for more than 2 years, a surreptitious onset, slow progression, and consistent lung involvement [2]. Chronicity can be heralded by lupus pernio, chronic eye involvement, bone localizations, and multi-organ involvement at onset [10]. Additionally, sarcoidosis can present in a silent, asymptomatic form, often discovered incidentally. Known as “the great mimicker”, sarcoidosis diagnosis requires a thorough medical history focused on exposure, physical examination, appropriate radiologic and pathologic studies, and the exclusion of other causes [11]. The Scadding scale, used for the radiological staging of lung involvement, categorizes chest X-ray abnormalities into four stages: (I) bilateral hilar lymphadenopathy; (II) bilateral hilar lymphadenopathy with pulmonary infiltrate; (III) pulmonary infiltrate alone; and (IV) fibrosis [6,9]. Sarcoidosis granulomas most frequently affect the mediastinal lymph nodes and lungs but can occur in virtually any organ. Forms with the most significant impact on quality of life and mortality include end-stage lung sarcoidosis, neurosarcoidosis, and cardiac sarcoidosis [2,6,9,12,13]. Regardless of whether the disease is acute or chronic, a biopsy confirming the presence of granulomas is required for diagnosis; however, the pathohistological identification of granulomas alone is insufficient without supportive radiological findings [9]. As of the date of manuscript preparation, comprehensive data pertaining to the incidence of sarcoidosis within the confines of our nation and the broader geographical expanse of this segment of Europe are conspicuously absent.

The principal objective of this investigation was to determine the incidence rate of sarcoidosis within the national context, concurrently providing a comprehensive overview of its various manifestations encompassing its clinical forms, biomarkers, and the therapeutic interventions administered. Additionally, this study endeavored to delineate the territorial disparities observed among patients diagnosed with sarcoidosis and receiving treatment at the Clinic of Pulmonology, University Clinical Center of Serbia, over the span of 23 years, from 2000 to 2023, including a thorough examination of their clinical characteristics.

## 2. Materials and Methods

In this retrospective study, a total of 2380 patients treated at the Clinic of Pulmonology, University Clinical Center of Serbia, in Belgrade, Serbia, were enrolled. All patients over 18 years of age who had a pathohistological confirmation of the diagnosis and underwent regular outpatient follow-ups between 2000 and 2023 were included in the study. Participation was voluntary and anonymized, with the required data being extracted from medical histories. For the facilitation of comparison, the study population was further divided into the following two groups: patients with acute sarcoidosis (1126 patients) and patients with chronic sarcoidosis (1254 patients), with identical data parameters collected from both groups. Prior to the start of the study, the study was approved by the Collegium of Clinic of Pulmonology, University Clinical Center of Serbia, and written informed consent was obtained from each participant.

### 2.1. Diagnostic Methodology

The diagnosis of sarcoidosis was established based on a combination of clinical, radiological, and histopathological criteria, adhering to the ATS/ERS/WASOG standards. Clinically, patients were evaluated for symptoms commonly associated with sarcoidosis, such as erythema nodosum, bilateral hilar lymphadenopathy, and pulmonary involvement.

A thorough medical history and physical examination were integral to the initial diagnostic approach. Radiologically, sarcoidosis was primarily identified through chest X-rays and high-resolution computed tomography. All patients had histological confirmation through tissue biopsies obtained via bronchoscopic, transbronchial, or needle techniques. All patients also underwent additional confirmatory testing, including serum calcium levels, angiotensin-converting enzyme (ACE) levels, pulmonary function tests, and bronchoalveolar lavage (BAL), to rule out any differential diagnoses.

### 2.2. Statistical Methodology

Descriptive statistics were computed for baseline demographic and clinical characteristics, as well as treatment outcomes. Graphical and mathematical methods tested the normality of distribution. Continuous variables were presented as means with standard deviations, while categorical variables were presented as numbers and percentages. Differences between groups were analyzed using Student’s *t*-test for continuous variables and Pearson’s chi-squared test for categorical variables. The significance level was set at 0.05, and all testing was two-sided. Statistical analysis was performed using IBM SPSS Statistics for Windows, version 21.0. (Armonk, NY, USA).

## 3. Results

Baseline clinical characteristics according to the form of disease are presented in Table 1.

**Figure 1:**
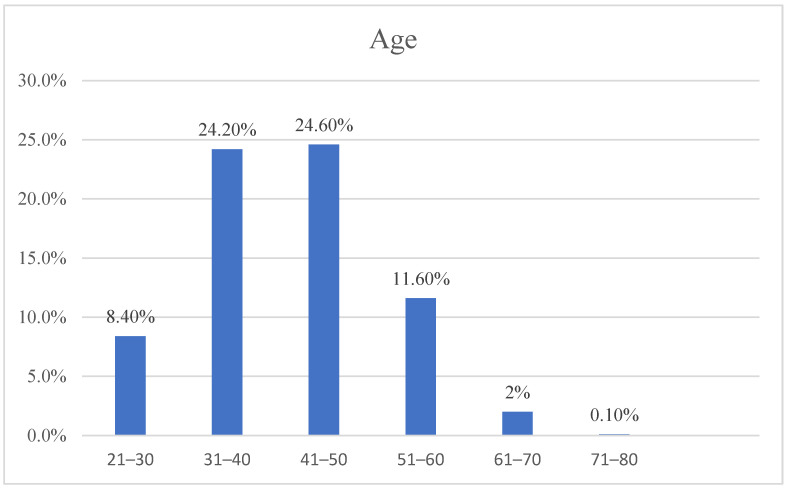
Age categories.

The acute form group consisted of 1126 patients, of which 395 (35.1%) were male and 731 (64.9%) were female. The chronic form group comprised 1254 patients, including 399 (31.8%) males and 855 (68.2%) females. The age distribution, presented in Table 1, displays an average age of patients of 46.8 years. Almost half of the patients (48.8%) were aged between 31 and 60 years (Figure 1).

**Figure 2:**
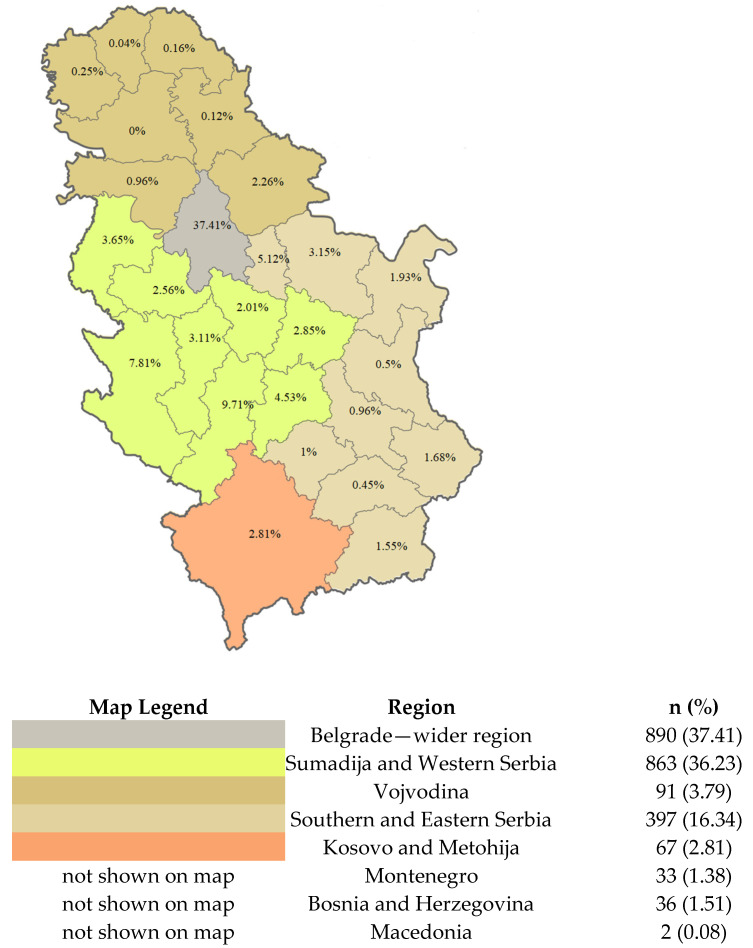
Geographical distribution of patients by region.

Patients with the chronic form were significantly older (*p* < 0.001). The geographical distribution of patients in Serbia is presented in Figure 2, with the majority of patients, 1465 (68.8%), residing in central and western Serbia, while only 97 (4.9%) patients came from the northern part of the country. 

Smoking status and professional exposure to the most common air pollutants are shown in Table 1, with no statistically significant differences observed between patients with acute and chronic sarcoidosis. Furthermore, with regard to extrapulmonary forms, all forms were more common in the chronic form to the level of statistical significance (Table 1).

Table 2 details the radiological findings in our patients. A total of 1668 patients had Stage I sarcoidosis according to the Scadding scale. This finding was present in 92.5% (1041) of patients with acute sarcoidosis, whereas only 50% (627) of patients with chronic disease had Stage I sarcoidosis. Conversely, a higher prevalence of Stage III and IV sarcoidosis was observed in patients with chronic disease, with 7.9% (101) of patients from the chronic form group having Stages III and IV compared to only 0.4% (5) of patients with acute disease. High statistical significance was noted in these differences (*p* < 0.001).

Regarding biomarkers, we selected the following standardized biomarkers used in routine diagnostics and treatment evaluation: angiotensin-converting enzyme (ACE), chitotriosidase (CHT), and calcium in 24 h urine (Table 3). We categorized patients based on whether these markers were elevated. Elevated values of CHT and calcium in 24 h urine were more common in patients with the chronic form, with these differences being statistically significant (*p* = 0.01 and *p* < 0.001, respectively).

## 4. Discussion

Sarcoidosis is an idiopathic granulomatous multisystem disease with a highly variable clinical presentation and an unknown direct cause that calls for further investigation. Our investigation into sarcoidosis within the Serbian population over a 23-year period has yielded insights into this multifaced disease, with patients presenting with a broad spectrum of clinical manifestations and outcomes. In our study, we uncovered the distinct demographic, clinical, and radiological patterns observed in our population. While the direct cause of sarcoidosis remains unknown, our study may further point us in the direction of uncovering specific factors that can play a potential role [2].

While sarcoidosis affects all populations regardless of age and ethnicity, epidemiological studies have noted variations among different populations. Notably, a study from a health maintenance organization in the USA showed that the age-adjusted annual incidence was 10.9 per 100,000 among Caucasian Americans and 35.5 per 100,000 for African Americans [14]. Another study yielded similar results where Ducheman et al. noted that a higher incidence was observed among the Scandinavian and African American populations when compared to the Caucasian population [15]. In a study examining the U.S. veteran population, the incidence and prevalence of sarcoidosis were reported to be significantly increased compared to the general population [16]. Furthermore, studies by Seedahmed et al. and Rossides et al. also pointed out a predominance among the African American population, female sex, history of tobacco use, and specific military service branches [16,17]. These findings highlight the potential role of environmental exposures related to occupation that can be attributed to the development of sarcoidosis. Similarly, in our study, we also observed a predominance in the female population for both chronic and acute forms. The gender disparity observed across multiple studies calls for future studies to investigate potential pathophysiological differences that may exist.

Furthermore, in our cohort, we also observed a statistical difference in the average age between patients presenting with chronic and acute forms, with a tendency for chronic forms to be more common in older patients (*p* < 0.001). These findings can be suggestive of age as a potential factor in the progression or clinical manifestation of sarcoidosis but also may suggest a latency in the development of sarcoidosis from the time of exposure. The further analysis of our cohort revealed the highest incidence among the working-age population, 31–60 years of age, further indicating that there could be an occupational risk but also signifying the significant socioeconomic implications of sarcoidosis. Notably, nearly half of our cohort, 48.8%, belonged to this age group. In our study, we took into account occupational exposures known to cause different respiratory illnesses, such as moldy hay, pine pollen, brick dust, beryllium, asbestos, coal dust, and silica. However, we found no statistically significant difference between patients with and without exposure. Even though previous studies have shown that there are potential risk factors for the development of sarcoidosis, there were no specific links identified, and therefore, we suggest that multiple factors play a role with a synergistic effect compounded over a period of time [18].

With regard to geographical distribution, we found that the majority of cases were concentrated in the central and western regions, including the capital, which could be suggestive of environmental factors or genetic predispositions in these areas. Of our study, nearly two-thirds of cases were in this area. The lowest number of cases in our cohort were from the northern region.

Since sarcoidosis is more prevalent in the mountainous regions, the distribution observed in our cohort is somewhat expected. However, when examining global estimates across Europe, there is an increase in incidence in the northern regions [4]. This may be, in part, explained by the varying terrain and environments. It is hypothesized that sarcoidosis may be linked to different microbiological and botanical biotypes present in the mountainous regions compared to the plains. Notably, the northern part of Serbia, where we observed the lowest number of cases, is a part of the Pannonian plain, which also further supports this hypothesis [2]. These findings can further imply the potential interplay of environmental factors and climate or even protective factors. It should be noted that this discrepancy can also possibly be attributed to underdiagnosis due to differences in access to healthcare. In addition, given that our study took place in a center that is located in the central part, there exists a bias of patients that tend to deviate towards the center despite being the University Clinical Center that is responsible for the whole country.

With regard to radiological findings, as categorized by the Scadding scale, there is a stark contrast revealed regarding disease progression between acute and chronic forms. Namely, 92.5% of acute sarcoidosis patients presented with Stage I disease, which is generally associated with a favorable prognosis. However, it is important to note that this stage does not guarantee non-progression, as some patients may still advance to more severe stages. Conversely, patients with chronic sarcoidosis exhibited a higher prevalence of more advanced stages III and IV, further highlighting the progressive nature and potentially severe pulmonary involvement associated with chronic sarcoidosis.

This discrepancy can be explained by the prolonged inflammation present in chronic sarcoidosis, which can lead to the destruction of pulmonary parenchyma and the proliferation of connective tissue. The prognostic value of the Scadding scale is important to note, as stage I disease is associated with a greater than 90% chance of resolving radiographic findings within 2 years, whereas for patients with stage III sarcoidosis, the percentage is less than 30% [19]. Our findings highlight the importance of early diagnosis and intervention to mitigate the long-term morbidity of this debilitating disease.

While there are still no universal criteria for the diagnosis of sarcoidosis, diagnosis relies heavily on the following three main criteria: presentation, the confirmation of non-necrotizing granulomas on histological examination, and the exclusion of differential diagnosis [2,20]. Some standard biomarkers that are routinely measured are the angiotensin-converting enzyme (ACE), chitotriosidase (CHT), and calcium levels. CHT has been shown to be the most selective, both compared to other biomarkers as well as in an acute vs. chronic comparison [21]. In our study, when analyzing the abovementioned biomarkers (ACE, CHT, and calcium in 24 h urine), we observed statistically significant elevations in CHT and calcium levels in chronic sarcoidosis patients compared to acute sarcoidosis patients (*p* < 0.001). Our findings are in alignment with previous studies, which have shown increased calcitriol concentrations in patients with sarcoidosis [22]. Notably, studies have also shown that the increased production of 1.25(OH) 2 D 3 has also been found to be responsible for calcium homeostasis disruption in sarcoidosis [22,23]. Calcium and CHT are considered to be indicators of disease activity, and these findings further suggest a plausible etiological suggestion of the disruption of calcium homeostasis, which is a complication of sarcoidosis that requires careful management. It is important to note that, unlike other biomarkers, we did not observe significant elevations in ACE. This could be explained by the fact that nearly a quarter of our patients had arterial hypertension treated with ACE inhibitors, and thus, the usage of ACE inhibitors could have led to false negative results.

With regard to extrapulmonary manifestations, these tended to be more common in patients with chronic sarcoidosis, highlighting the systemic nature of the disease and its known effects on virtually any organ system. Studies have shown that the skin is the second most common organ involved in sarcoidosis [24]. Most frequently, cutaneous manifestations can be seen as maculopapular, hypopigmented, or hyperpigmented lesions, ulcers, pustules, and subcutaneous nodules [25]. The results of our study are in alignment with previous studies as the majority of our patients who had an extrapulmonary form of sarcoidosis had sarcoidosis of the skin (187/855), followed by ocular (173/855) and neurosarcoidosis (108/855). In our cohort, we also observed extrapulmonary sarcoidosis with those of the skin, eye, brain, heart, liver, spleen, and bone lesions being the most common among patients suffering from the chronic form of the disease compared to those with acute sarcoidosis. These findings further point toward the necessity for comprehensive clinical evaluations and a multidisciplinary approach to management, particularly in chronic cases where the disease burden is likely to be more extensive.

### Limitations

While our study provides significant insights into the epidemiological and clinical profiles of sarcoidosis in Serbia, it does possess inherent limitations associated with its retrospective design. Given that all data were sourced from a single institution, the findings may not be generalizable to other populations or geographic settings, potentially limiting their applicability despite involving patients from various regions. The retrospective nature of this study might also introduce information bias, as it relied on pre-existing medical records, which could vary in accuracy and completeness. Additionally, the study spanned over two decades, a period during which diagnostic technologies and criteria evolved significantly, possibly affecting consistency. The absence of a control group further constrained our ability to draw definitive conclusions about the specificities of sarcoidosis compared to other pulmonary conditions. Variations in the frequency and thoroughness of patient follow-ups could also introduce further variability into the data, influencing the robustness of the outcome measures.

## 5. Conclusions

While it is evident that sarcoidosis is a rare disease in Serbia, the findings of our comprehensive study highlight the distinct epidemiological geographic distribution of sarcoidosis within the country. Our research also further delineates the clinical profiles of patients within this region, revealing pronounced variability in disease prevalence between different topographical regions. This variability suggests the potential influence that environmental and genetic factors may have. Notably, our study distinguishes between acute and chronic forms of sarcoidosis, highlighting their epidemiological differences. Future research is warranted to delve into the underlying mechanisms that may be responsible for the variances observed, which could lead to more targeted interventions and management strategies for sarcoidosis.

## Figures and Tables

**Table 1:** Study population characteristics.

	Mean Age ± SD (Minimum–Maximum Value)			
Age	46.8 ± 11.7 (21.0–84.0)			
	Total (*n* = 2380)	Acute (*n* = 1260)	Chronic (*n* = 1254)	*p*-Value
Smoker	794 (33.4)	395 (35.1)	399 (31.8)	0.092
Non-smoker	1586 (66.6)	731 (64.9)	855 (68.2)	
Previous smoker	423 (17.8)	210 (18.7)	213 (17)	0.066
Occupational exposure (yes)	1729 (72.6)	824 (73.2)	905 (72.2)	
Moldy hay	228 (9.6)	92 (8.2)	136 (10.8)	
Pine pollen	522 (21.9)	259 (23)	263 (21)	0.232
Brick dust	76 (3.2)	44 (3.9)	32 (2.6)	0.06
Beryllium	61 (2.6)	33 (2.9)	28 (2.2)	0.282
Asbestos	153 (6.4)	69 (6.1)	84 (6.7)	0.571
Coal dust	32 (1.3)	14 (1.2)	18 (1.4)	0.685
Silica	36 (1.5)	17 (1.5)	19 (1.5)	0.991
Other	164 (6.9)	85 (7.5)	79 (6.3)	0.23
Extrapulmonary forms				
Eye	173 (7.3)	14 (1.2)	159 (12.7)	<0.001
Heart	61 (2.6)	3 (0.3)	58 (4.6)	<0.001
Liver	61 (2.6)	1 (0.1)	60 (4.8)	<0.001
Spleen	60 (2.5)	2 (0.2)	58 (4.6)	<0.001
Bone marrow	11 (0.5)	1 (0.1)	10 (0.8)	
Bones	30 (1.3)	1 (0.1)	29 (2.3)	<0.001
Skin	187 (7.9)	12 (1.1)	175 (14)	<0.001
Neurosarcoidosis	108 (4.5)	6 (0.5)	102 (8.1)	<0.001
Other	164 (6.9)	10 (0.9)	154 (12.3)	<0.001

All data are presented as *n* (%) or mean ± SD (minimum–maximum) depending on variable type.

**Table 2:** Radiological findings on chest radiography by Scadding classification.

		Total (*n* = 2380)	Acute (*n* = 1126)	Chronic (*n* = 1254)	*p*-Value
Stage	0	6 (0.3)	1 (0.1)	5 (0.4)	<0.001
	1	1668 (70.1)	1041 (92.5)	627 (50)	
	2	600 (25.2)	79 (7)	521 (41.5)	
	3	95 (4)	5 (0.4)	90 (7.2)	
	4	11 (0.5)	0 (0)	11 (0.9)	
Atypical finding		29 (1.2)	7 (0.6)	22 (1.8)	0.012

All values in the table are presented as *n* (%).

**Table 3:** Biomarkers in sarcoidosis.

	Clinically Significant Value	Total (*n* = 2380)	Acute (*n* = 1126)	Chronic (*n* = 1254)	*p*-Value
Angiotensin converting enzyme	>56	900 (40)	426 (40.5)	474 (39.6)	0.665
Calcium in 24 h urine	>7.5	201 (9.4)	70 (7)	131 (11.6)	<0.001
Chitotriosidase	>156	658 (69.9)	317 (76.4)	341 (64.8)	0.001

## Data Availability

The data that support the findings of this study are available from the corresponding author (M.S.) upon reasonable request.
